# Development of early warning and rapid response system for patients with novel coronavirus pneumonia (COVID-19): A research protocol

**DOI:** 10.1097/MD.0000000000021874

**Published:** 2020-08-21

**Authors:** Hua Zhou, Huibin Huang, Xiaolei Xie, Jiandong Gao, Ji Wu, Yan Zhu, Wei He, Jingyuan Liu, Ang Li, Yuan Xu

**Affiliations:** a Department of Critical Care Medicine, Beijing Tsinghua Changgung Hospital, School of Clinical Medicine; b Center for Healthcare Service Research, Department of Industrial Engineering; c Center for Big Data and Clinical Research, Institute for Precision Medicine; d Department of Electronic Engineering, Tsinghua University; e Department of Critical Care Medicine, Beijing Tongren Hospital; f Department of Critical Care Medicine, Beijing Ditan Hospital, Capital Medical University, Beijing, China.

**Keywords:** cohort study, COVID-19, critically ill, deterioration, early warning, rapid response

## Abstract

**Background:**

Coronavirus disease 2019 (COVID-19) has caused serious damage to public health. COVID-19 has no vaccine or specific therapy; its mortality rate increases significantly once patients deteriorate. Furthermore, intensive monitoring of COVID-19 is limited by insufficient medical resources and increased risks of exposure to medical staff. We therefore aim to build an early warning and rapid response system (EWRRS) to address these problems.

**Method:**

The research is designed as a prospective cohort study, to verify a dynamic and interactive evaluation system; it includes patient self-reporting, active monitoring, early alarming and treatment recommendations. Adult patients diagnosed with COVID-19 will be recruited from Sept 2020 to Aug 2021 at a tertiary contagious hospital. Patients with life expectancy <48 hours, pregnant or lactating, in immunosuppression states or end-stage diseases will be excluded. The intervention is implementation of EWRRS to detect early signs of clinical deterioration of COVID-19 patients, to provide timely and efficient treatment suggestions by the system. EWRRS can determine the classification and interactive evaluation of patient information; the determination is based on the application of 3 different scenario modules, separately driven by patients, nurses, and physicians. The primary outcome is change in disease severity category after treatment. Secondary outcomes include the proportion of patients with different disease severity types; critical deterioration events; patients who had unplanned transfers to an intensive care unit (ICU) and required critical care interventions; intervals from warning to implementation of clinical interventions; hospital mortality; length of ICU and hospital stay; workload of medical staff and risks of exposure to COVID-19.

**Discussion:**

Our hypothesis is that EWRRS provides an example of an early identification, warning, and response system for COVID-19. In addition, EWRRS can potentially be extended to use as a grading metric for general critically ill patients in an ICU setting.

## 1 Introduction

Since December 2019, a novel coronavirus, COVID-19, has been identified from a cluster of contagious cases that mainly presented as pneumonia. The outbreak has been spreading around the world. Through the end of June, confirmed coronavirus cases in China were 85 thousand; reported confirmed coronavirus cases in the rest of the world reached 9.8 million, with more than 49 million deaths. According to current reported data, the overall mortality rate due to COVID-19 was approximately 5%. The mortality rate of severe cases can be much higher, varying from 22% to 62%.^[1–3]^

COVID-19 patients present a wide range of signs and symptoms—fever, dry cough, fatigue, shortness of breath, dyspnea, palpitation, persistent pain or pressure in the chest, myalgia, diarrhea, etc. Severe symptoms such as short breathing and lower oxygenation might appear around 10 days after initial symptoms. More severely impacted patients might progress quickly to Acute Respiratory Distress Syndrome, requiring intubation and mechanical ventilation. Further worsening might lead to septic shock and organ dysfunction. Consequently, close and frequent monitoring of symptoms and progress is required for COVID-19 patients.

In order to prevent the spread of the disease, many countries built mobile cabin hospitals to isolate and collectively treat patients. Severe and critical cases should be identified for early intervention: it is essential for improving survival probability and reducing mortality. However, due to the shortage of healthcare staff and limited medical resources, it is very difficult to fulfill such a large workload of monitoring and treatment tasks.

Consequently, we plan to build a dynamic and interactive reporting system, including patient self-reporting, active monitoring, prompt alarming, and treatment recommendations. This Early Warning and Rapid Response System (EWRRS) can provide a platform for patient data exchange by establishing an information processing system, thus engaging patients to participate in self-assessment. The system will report signs, symptoms, and physical examination in real time. All this information will be used for early identification of disease progression, to trigger recommendations for further medical care.

**Figure 1:**
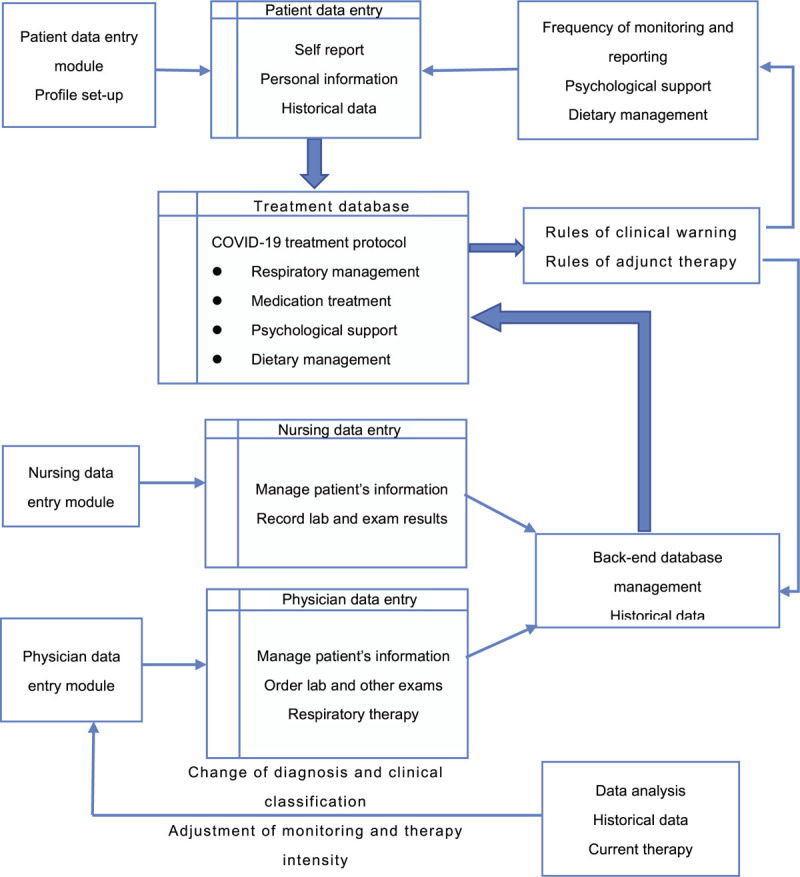
COVID-19 Early Identification, Warning, and Response System design flow chart: a continuous novel coronavirus pneumonia self-reporting cyclic feedback management system incorporating “identify-feedback-intervene-evaluate-reidentify.”

EWRRS is equipped with a complete data processing and evaluation system; it evaluates cut-off values for a variety of clinical parameters. An alarm module setting, applied to coronavirus pneumonia clinical signs/symptoms and clinical severity classifications, will warn when test measures are in excess of cut-off values, predicting when patient condition is becoming more severe. The alarm includes 2 levels, a lower level of warning and higher level of alarming. For instance, critical warning starts when heart rate and blood pressure fluctuate more than 20% from baseline. The lower warning level means oxygen saturation from pulse oximetry (SpO_2_) is more than 2% lower than 93%. If SpO_2_ further decreases, even with supplemental oxygen, it implies that the patient may have a severe course of the disease. The higher level of alarming then will be triggered. As a result, treatment recommendations can be made by physicians and nurses according to patient risk classification and warning level, considering the individual hospital's situation and capacities. These recommendations may include increasing the frequency of monitoring, change of oxygen therapy, advancing respiratory therapy, or transfer of patients to ICU. EWRRS can determine the classification and interactive evaluation of patient information based on the application of 3 different scenario modules—patient, nurses, and physician (Fig. 1): *Patient-driven data evaluation module.* Once admitted, each patient will be requested to log into this system via a predesigned smartphone app. Next, the system will prompt patients to enter their demographic information; it then will guide the self-evaluation and documentation of signs and symptoms under physician order. Subsequently, physicians will provide initial assessment, clinical classification and offer preliminary treatment recommendation according to patients’ current medical conditions. For mild/moderate cases, at least 2 daily self-reports of vital signs are required. The system will offer professional feedback, including suggestions on treatment and recovery to patients. *Nursing-driven data evaluation modul*e. The system will automatically load patient self-reported information, which becomes part of medical record, into this module. If there is any identified abnormal data from vital signs and lab tests, or overdue self-reporting, the system will trigger a warning for nursing to intervene. This could include close observation, more frequent monitoring, dietary management, and/or psychological support, etc. *Physician-driven data evaluation module*. Similar to the nursing data entry module, this module will include all of the key administrative clinical data relevant to that person, including demographics, nursing and physician progress notes, medical problems, medications, lab tests, and results from various diagnostic exams. The system will integrate all the information to identify patients with abnormal clinical presentation, project the disease progression, and provide a priority list of patients that require physician attention. According to different levels of notice (warning or alarming), physicians will decide if more frequent clinical monitoring, checks on blood gas, and alternative therapies are required. After these interventions, the system will reevaluate patients’ updated clinical data, reclassify patients, and adjust treatment plans if needed. This system will incorporate continuous feedback management: identify–feedback–intervene–evaluate–reidentify.

The COVID-19 EWRRS will facilitate early identification of patients that potentially may progress from mild or moderate to severe or critical illness. Moreover, the development of this system will also be helpful for identification and response for future epidemic disease crises and other public health emergency events.

## 2 Methods

The protocol is reported according to the Standard Protocol Items: Recommendations for Interventional Trials Checklist.^[4]^ Our protocol has been registered on the Chinese Clinical Trial Registry (http://www.chictr.org.cn/index.aspx). The registration number was ChiCTR2000034680.

### 2.1 Study design and aims

This is a stepped-wedge prospective mixed methods study exploring the feasibility of EWRRS, which is an interactive, multimodal screening program to support the early identification of potential patients who may progress from mild or moderate to severe or critical illness. The detailed study aims:

1. To improve the detection of early signs of clinical deterioration of COVID-19 patients and provide real-time data to the entire clinical team, thus improving team situation awareness of patient deterioration.
2. To provide timely and efficient treatment suggestions for COVID-19 patients through the rapid response system, thereby promoting active management of deterioration, stabilizing patient conditions early and avoiding the progression of deterioration to a more serious degree.
3. To reduce the workload and risks of occupational exposure of medical personnel during the epidemic through interactive, multimodular processes.
4. To identify the COVID-19 progression patterns and characteristics through big data analysis, thus providing an example of EWRRS for future outbreaks of public health events. Moreover, such a system can potentially be extended for use as a grading EWRRS, to respond to deteriorating patients outside of an ICU setting.

### 2.2 Study population and setting

Patients are eligible for this study if they were diagnosed with COVID-19 according to the New Coronavirus Pneumonia Prevention and Control Program (7th edition), published by the National Health Commission of China.^[5]^ Inclusion criteria comprise the following: adult patients (18 years or older) with clear epidemiological history; fever and/or respiratory symptoms; normal or decreased white blood cell count and/or lymphocyte count; high degree of suspected imaging characteristics of novel coronavirus pneumonia; positive results of SARS-CoV-2 nucleic acid detection and serum-specific antibodies; ability to provide a signed informed consent form. Patients were excluded if known life expectancy was less than 48 hours, pregnant or lactating, diagnosed with end-stage diseases, or in immunosuppression states.

Clinical Classification of the COVID-19 patients is according to their disease severity upon admission: *Mild cases*: The clinical symptoms are mild and no pneumonia manifestations can be found in imaging. *Moderate cases*: Patients have symptoms such as fever and respiratory tract symptoms, etc. and pneumonia manifestations can be seen in imaging. *Severe cases*: Adults who meet any of the following criteria: respiratory rate ≥30 breaths /min; oxygen saturation ≤ 93% at a rest state; arterial partial pressure of oxygen (PaO_2_)/oxygen concentration (FiO_2_) ≤ 300 mm Hg; patients with > 50% lesion progression within 24 to 48 hours in lung imaging. *Critical cases*: Adults who meet any of the following criteria: occurrence of respiratory failure requiring mechanical ventilation; presence of shock; other organ failure that requires monitoring and treatment in the ICU.

*Hospital discharge criteria*: normal body temperature for more than 3 days; significantly improved respiratory symptoms, that is, no oxygen supplementation requirement, stable and normal vital signs for longer than 1 day; lung imaging showing obvious absorption and resolution of acute infiltrates; negative results of the nucleic acid test for SARS-CoV-2 two times consecutively, with at least a 1-day interval between tests.

Patient recruitment duration will last for a year, from September 2020 to August 2021. During the study period, patients diagnosed with COVID-19 will be directly admitted or transferred to Beijing Ditan Hospital, which is the government designated COVID-19 hospital.

### 2.3 EWRRS procedures

**Figure 2:**
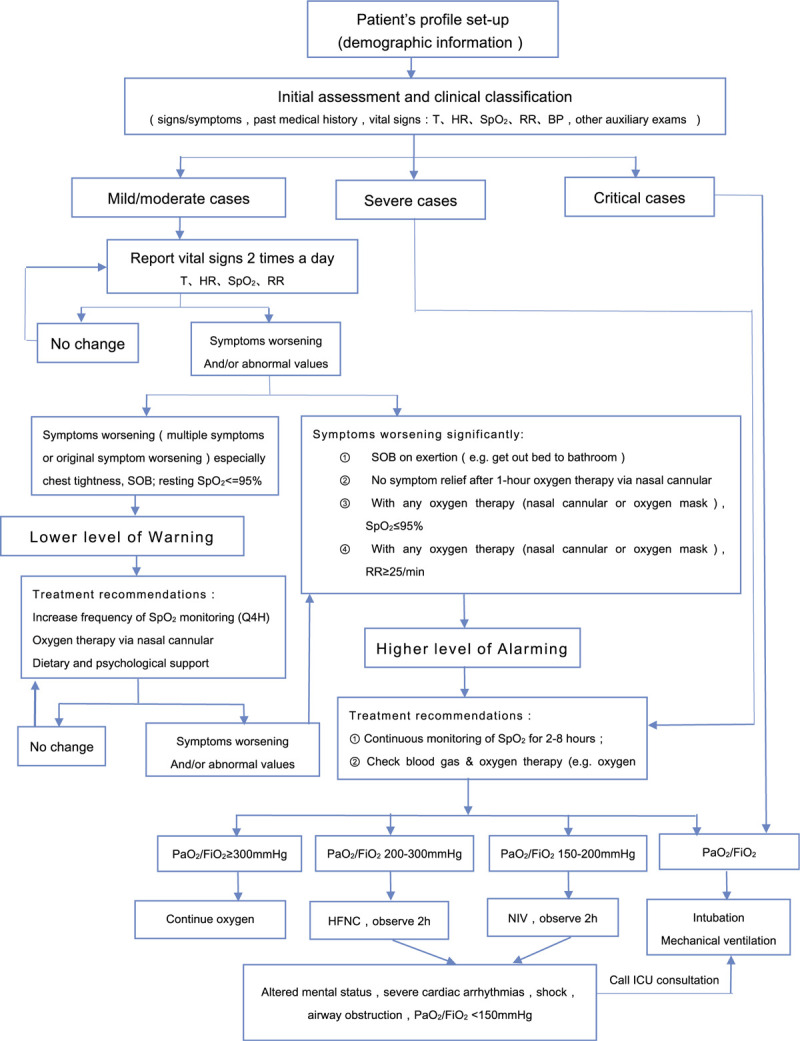
Algorithms for different levels of the warning system and corresponding treatment recommendations.

For recruited COVID-19 patients, the EWRRS will perform different levels of assessment for the patient alarm information, offering corresponding treatment recommendations (Fig. 2). The details in EWRRS procedures are as follows:

1. After admission, EWRRS performs an initial assessment of COVID-19 patient disease severity based on past medical history, clinical symptoms, vital signs, and other auxiliary examinations. It then classifies them into 3 clinical classifications: mild/moderate, severe, and critical cases. Similarly, during the subsequent treatment, EWRRS will reclassify patients according to the results of updated patient clinical data, if needed.
2. For mild/moderate cases, EWRRS will provide “self-reported vital signs” twice to 3 times per day. During treatment, these cases may present mild abnormalities in vital signs or values in lab tests—chest tightness, shortness of breath, resting SpO_2_ less than 95% etc.; these will be regarded as “lower level warning” by the system. The system will then trigger a warning for nursing intervention, with treatment recommendations. These include “Increase frequency of SpO_2_ monitoring (every 4 hours),” “Oxygen therapy via nasal cannular,” and “Dietary and psychological support.”
3. Cases where patient symptoms and lab values grow worse and meet the following standards: shortness of breath on exertion (e.g., getting out bed to bathroom); no symptom relief after 1-hour oxygen therapy via nasal cannular; SpO_2_≤95% with any oxygen therapy (nasal cannular or oxygen mask); respiratory rate ≥25/min with any oxygen therapy (nasal cannular or oxygen mask); ROX≤4.88 with any oxygen therapy (nasal cannular or oxygen mask). These worsening symptoms will be identified as “higher level alarming” by the system. Meanwhile, these patients will be reassessed as severe cases. The system will synchronously trigger a warning for physicians to intervene. As a result, these severe cases will receive more monitoring of SpO_2_, blood artery gas check and various intensive respiratory supports according to their PaO_2_/FiO_2_, a blood gas parameter that reflects oxygenation status.
4. For severe cases—PaO_2_/FiO_2_ of ≥300 mm Hg; 200 to 300 mm Hg; and 150 to 200 mm Hg—treatment recommendations become “continue oxygen therapy,” “high-flow nasal cannula oxygen therapy, observe 2 hours,” and “noninvasive ventilation, observe 2 hours,” respectively. If these cases continue to deteriorate—altered mental status presented, severe cardiac arrhythmias, shock, airway obstruction, patients with PaO_2_/FiO_2_ <150 mmHg—they will be reevaluated as critical cases by the system; a treatment recommendation of “Call ICU consultation” would then be offered.

### 2.4 Outcome measurements

The primary outcome is change in the clinical classification of COVID-19 patients, including postintervention and treatment. The clinical experts will perform disease severity and final clinical classification assessment according to predetermined criteria.

Secondary outcomes include the proportion of patients with different disease severity types; critical deterioration events of patients during treatment, including total incidences and classification of each type of event; patients with unplanned transfers to ICU and requiring critical care interventions (mechanical ventilation, noninvasive ventilation, inotropes, and/or other organ-supported therapies) during treatment; time intervals from patient warning information received to implementation of clinical interventions; the severity of illness at unplanned transfer to ICU; hospital mortality; length of ICU and hospital stay; workload of physicians and nurses during study period; risks of exposure to potential COVID-19 patients.

### 2.5 Data collection and management

After admission, the system will record the patient demographic characteristics, age, gender, and past history. Signs and symptoms, physical examination results, laboratory tests, and imaging examination will be also recorded as baseline data. During the research period, the system will dynamically record all self-report and laboratory test results of the patient throughout the process. These specific parameters include signs and symptoms: dry cough, persistent chest pain, palpitation, myalgia, shortness of breath, dyspnea, etc.; vital signs: body temperature, heart rate, respiratory rate, SpO_2_, blood pressure; laboratory tests: white blood cell count, lymphocyte count, platelet count, C-reactive protein (CRP), transaminase (AST, ALT), bilirubin, creatinine, lactate dehydrogenase, troponin, D-dimer, blood gases, etc.; diagnostic radiology of the lungs: initial and follow-up imaging features of chest X-rays, chest computed tomography scans, and evaluation of dynamic imaging findings throughout the course of the disease; complications, such as shock, cardiovascular events, renal failure, etc. All the alarming information and levels that trigger EWRRS will also be completely recorded.

The data for all patients participating in the study will be securely stored in the EWRRS and exported to a specially designed database. It will be ensured that data is collected during the entire 24 hours and on all days of the week during the study.

### 2.6 Statistical analysis

We used the MedCalc statistical software, version 15.6.1 for Windows, and GraphPad Prism 7 in the present study. Categorical variables were expressed as numbers and proportions (%). Continuous variables were expressed by mean value ± standard deviation (SD) if the distribution of the original or transformed variable was normally distributed or by the median along with 25 to 75 interquartile ranges (IQR) if the distribution of the variable was normal. Proportions and means (SD) were compared by the Pearson *χ*^2^ test and Student *t* test, respectively. Means (IQR) were compared by the Wilcoxon test. A *P* value of <.05 was defined as statistically significant.

### 2.7 Trial management

#### 2.7.1 Coordination centers

The present research involved a collaboration of the Department of Critical Care Medicine, Beijing Tsinghua Changgung Hospital, and the Department of Electronic Engineering and Institute for Precision Medicine, Tsinghua University. Team members included clinical experts, statisticians and data analysts, and big data engineers. They were responsible for clinical research methodology and addressing key problems in the implementation of research.

#### 2.7.2 Quality control

Sites and researchers were monitored and inspected regularly by the Hospital's Clinical Research Supervision Committee, following the standard protocol throughout the study process.

### 2.8 Ethics and dissemination

Study patients or their authorized surrogates received sufficient explanation and time to sign the informed consent form before the trial. Researchers ensured that the study was conducted in accordance with the principles of the Declaration of Helsinki and the clinical trial quality management regulations in China. The study protocol has been approved by the Ethics Committee of Beijing Tsinghua Changgung Hospital (20262-0-01).

## 3 Discussion

With more and more patients affected, the global COVID-19 pandemic has become a significant public health emergency. The whole world now is fighting this pandemic, building mobile cabin hospitals to isolate patients, conducting intensive clinical investigation and research studies on confirmed cases. We must overcome staffing shortages due to the need to care for more patients, healthcare personnel exposures, and illness. The data processing and evaluation system is built to collect and evaluate clinical signs and symptoms, point-of-care testing and center lab test results and their progressive dynamic changes. It will assist clinicians in filtering high-risk patients among all isolated patients.

The 2019 novel coronavirus infection is an illness mainly targeting the respiratory tract. The initial symptoms might appear nonspecifically and can go unnoticed. More serious cases might present difficulty breathing and severe hypoxemia around 7 to 10 days after exposure to the coronavirus; they will progress quickly, and eventually require mechanical ventilation. The mortality of these severe patients can be as high as 88%.^[5]^ Some might only present shortness of breath or chest distress, described by low oxygen level exhibited without dyspnea, termed as silent hypoxemia. For dynamic monitoring, it is therefore essential to identify these potentially severe cases actively.

Attentive patients are observant of illness progression. This is the foundation for the success of self-reporting of signs and symptoms, with active participation in the process of care. Previously, we created a probability and severity risk assessment matrix based on clinical presentations and auxiliary examinations, to estimate the severity of ICU patients, the risk of thrombosis, and the readmission rate for heart failure patients, etc.^[6,7]^ Here as well, the identification of a high-risk group at an early stage will bring about timely intervention, thereby reducing the possibility of medical error and hospital burden.

Besides respiratory symptoms, other factors—shortness of breath, advanced age (age 75 or older), existing cardiovascular disease, diabetes mellitus, and other underlying diseases—will influence the prognosis of the novel coronavirus pneumonia.^[8]^ Similar to severe acute respiratory syndrome (SARS) and Avian Influenza, other factors have been linked to negative clinical outcomes for these patients: continuously decreasing lymphocyte count; progressively increasing inflammatory cytokines (such as interleukin, IL-6), C-reactive protein (CRP), lactate, lactate dehydrogenase.^[9,10]^ Many clinical observation studies reported coagulopathy, reduced platelet count, and elevated D-dimer among these patients. Among 1099 COVID-19 cases, a higher percentage of severe/critical cases presented low platelet count (≤150 × 10^9^ /L) than in mild/moderate cases (57.7% vs 31.6%);^[11]^ there was a significantly higher number of patients with D-dimer ≥0.5 mg/L in severe/critical cases (59.6% vs. 43.2%). These observations suggest a close relationship between coagulopathy and the severity and prognosis of this disease. Other concomitant symptoms and injuries manifested as abnormal liver function—such as increased troponin and bilirubin—and abnormal EKG presentations.^[12–14]^ The data processing and evaluation system will include all the clinical indices mentioned above; thereafter, it will set up different warning levels, to assist clinicians in assessing the severity and need to intervene. This system will advise different levels of monitoring and intervention, to monitor high-risk patients and provide treatment solutions. The result should be a significant improvement in early warning efficiency. At the same time, the system will harvest a database for an early predicting system, by incorporating the epidemiological characteristics.

The EWRRS will be significant in several respects: It provides an example of an early identification, warning, and response system for future outbreaks of public health events. It can be converted to a regular early warning system for hospital patients with emergency events in out of ICU. It helps to identify the disease (COVID-19) progression patterns and characteristic through big data analysis.

## Author contributions

Yuan Xu and Ang Li planned the study design and wrote the study protocol. Jiandong Gao, Ji Wu and Xiaolei Xie reviewed the study protocol. Huibing Huang, Yan Zhu, Wei He and Jingyuan Liu will recruit participants and collect data. Hua Zhou wrote the manuscript. All of the authors have read, commented on, and contributed to the submitted manuscript.
